# eHealth Literacy and Participation in Remote Blood Pressure Monitoring Among Patients With Hypertension: Cross-Sectional Study

**DOI:** 10.2196/71926

**Published:** 2025-07-31

**Authors:** Chinwe E Eze, Michael P Dorsch, Antoinette B Coe, Corey A Lester, Lorraine R Buis, Karen B Farris

**Affiliations:** 1 College of Pharmacy-Clinical Pharmacy Department, University of Michigan–Ann Arbor, 428 Church Street, Ann Arbor, MI, 48109, United States, 1 7346806587; 2 Department of Family Medicine, University of Michigan, Ann Arbor, MI, USA

**Keywords:** electronic health literacy, remote blood pressure monitoring, telemonitoring, hypertension, blood pressure, patients, digital health literacy

## Abstract

**Background:**

The ability to participate in digital health services such as remote blood pressure monitoring needs digital skills and knowledge known as eHealth literacy (e-HL). However, e-HL is rarely studied among those participating in remote blood pressure monitoring (RBPM).

**Objective:**

This study assessed e-HL levels among participants with hypertension and determined the e-HL domains that predict participation in RBPM. This study provides important focus areas to increase RBPM participation.

**Methods:**

This study was a quantitative, cross-sectional survey of people with hypertension in the United States. The survey included demographics, RBPM participation questions, and the e-HL questionnaire (eHLQ) for assessment of e-HL. The eHLQ is a 35-item, 7-domain validated questionnaire including the (1) ability to process information, (2) engagement in own health, (3) ability to actively engage with digital services, (4) feel safe and in control, (5) motivated to engage with digital services, (6) access to digital services that work, and (7) digital services that suit individual needs. The eHLQ item scores range from 1 to 4, and the higher the score, the higher the e-HL status. Descriptive statistics were used to describe the participants’ demographics and e-HL status. *χ*^2^ tests were used to compare participants’ characteristics between RBPM and nonRBPM groups. The Mann-Whitney *U* test compared the e-HL domain scores in RBPM and nonRBPM groups. Firth logistic regression was used to predict participation in RBPM. The dependent variable was participation in RBPM. The independent variables were demographics and e-HL domains.

**Results:**

A total of 507 people with hypertension participated in the survey. Sixty participants were currently participating in RBPM, giving a prevalence of 11.8% (60/507). The mean age of RBPM participants was 46.2 (SD 14.7) years and nonRBPM was 62 (SD 13.7) years (*P*<.001). The e-HL scores in all 7 domains were significantly higher for the RBPM group than the nonRBPM group. Among the e-HL domains, higher scores in digital services that suit individual needs (domain 7) were the only predictor of RBPM participation (adjusted odds ratio 2.84, 95% CI 1.002‐8.84) adjusted for age, sex, and race.

**Conclusion:**

Digital services that are tailored to individual patients’ needs are more likely to result in participation in RBPM.

## Introduction

### Background

Hypertension has remained a public health concern in the American adult population. Hypertension, defined as systolic blood pressure (BP) ≥130 mm Hg or diastolic BP ≥80 mm Hg or both, has a prevalence of 46.7%, and control (systolic BP <130 mm Hg and diastolic BP <80 mm Hg) is achieved in just 25.7% of adult Americans with hypertension diagnosis [1]. BP telemonitoring, also known as remote blood pressure monitoring (RBPM), has been identified as a viable means to engage and help patients with hypertension achieve control [2-4]. It involves the electronic transmission of self-measured BP from the comfort of a patient’s home to their health care provider’s office or secure website that health care providers can access. RBPM is currently the readiest implementable digital solution to hypertension control [5]. To successfully engage in RBPM, the patient is, however, required to have the skill and knowledge needed to operate the technology. This concept is known as eHealth literacy (e-HL) [6-8], and it is an important part of RBPM participation.

Over the years, several models and scales of e-HL have been proposed to measure e-HL [6-20]. There is currently no gold standard for e-HL measurement. For this project, the eHealth literacy questionnaire (eHLQ) [15] developed from the eHealth literacy framework (e-HLF) [6] was used because it has input from relevant stakeholders and the content acknowledges the role of the individual and the eHealth systems. The robustness of this framework makes it a viable tool to understand why patients with hypertension may or may not be engaging in technologies proven to improve BP control.

The e-HLF is a model of e-HL put forward by Norgaard et al [6] It was developed by systematic inductive methods involving inputs from patients with chronic health conditions, IT experts, health professionals, and eHealth professionals to capture all the elements that may impact individuals’ decisions to use eHealth for their health management. The e-HLF posits e-HL as a function of external observable and internalized traits of individuals and the eHealth systems, and the interactions between them. They thus proposed 7 dimensions or domains of e-HL including the (1) ability to process information, (2) engagement in own health, (3) ability to actively engage with digital services, (4) feel safe and in control, (5) motivated to engage with digital services, (6) access to digital services that work, and (7) digital services that suit individual needs. The corresponding eHLQ [15] developed from e-HLF provides an essential tool to measure a patient’s e-HL status. eHLQ considers a patient’s health knowledge and technology skills, motivation to engage in technology, access to technology, and health data security concerns. eHLQ reflects most of the patient-related factors that may influence RBPM engagement [15]. The assessment of a patient’s e-HL status is essential to ensure maximum benefit from any health digital services, including RBPM.

However, patients’ e-HL status is seldom assessed in eHealth intervention studies [21,22] and RBPM studies are no exception. The few eHealth management studies involving patients with hypertension either used a nonvalidated tool [23] or the eHealth Literacy Scale (eHEALS) to measure e-HL status [24-27]. A small number of these studies are focused on RBPM [23,25]. eHEALS [7] centers mainly on patients’ ability to use the internet and does not have the robustness of eHLQ. Moreover, studies on RBPM are mainly intervention studies and do not reflect daily life routines among people with hypertension. Data on concurrent assessment of RBPM participation and e-HL status as predictors are also limited. Studies focused on RBPM using eHLQ are therefore warranted.

### Objective

This study aimed to assess e-HLF domains that predict participation in RBPM among adults with hypertension. We hypothesized that participation in RBPM is associated with patients’ e-HL status.

## Methods

The data for this project were collected from the respondents with hypertension who participated in the project “Behavioral Factors Related to Participation in Remote Blood Pressure Monitoring Among Adults with Hypertension: Cross-sectional Study” published in *JMIR Formative Research* [28]. Therefore, most of the methods and the demographics results below were reproduced from that paper. For this study, RBPM participation is defined as participants’ remote transfer of self-measured BP to their health care providers via several methods including SMS text messaging, phone calls, emails, eHealth records, and automatic transmission.

### Design

This study was a quantitative cross-sectional survey of patients with hypertension in the United States.

### Participants

The inclusion criteria were patients aged ≥18 years who self-reported hypertension diagnosis, had at least one prescription hypertension medication, understood the English language, and were willing to participate. Exclusion criteria included active cancer, diagnosis of cognitive impairment, or having been to the intensive care unit in the past 6 months. We used the exclusion criteria because people with active cancer, cognitive impairment, or who were recently in the intensive care unit were more likely to be closely monitored by their health care providers and may not provide the general RBPM practice obtainable in the hypertension population.

### Sample Size

With 47.3% adult population with hypertension in the United States in 2021 [29], using 5% type 1 error (*P*=.05), the minimum sample size required to estimate participation in RBPM was 383 participants [30]. A minimum of 500 sample size has been recommended for detecting differences between the sample estimates and the population in observational studies involving logistic regression [31]. We stopped recruitment as soon as possible when we reached a sample size of 500. Therefore, 507 participants with hypertension were recruited using the quota sampling explained in the Recruitment section.

### Recruitment

Participants were recruited using an online Qualtrics panel [32]. Qualtrics panel members are real people whose names, addresses, and date of birth have been validated. Qualtrics recruits them from all over the United States to participate in surveys. Based on the preliminary study of a secondary analysis of the 2018 Health Information National Trends Survey (HINTS) 5 Cycle 2, age and education were associated with eHealth information seeking among respondents with hypertension. Therefore, a priori quota sampling based on age and educational levels was used. To ensure adequate representation within the age and education groups, the following proportions from our preliminary study were used: less than 50 years (15%), 50‐74 years (64%), 75 years and above (21%), less than college education (32%), some college education (34%), and college graduate and above (34%). The study, extent of participation, and incentive for participation were described to the participants meeting the inclusion criteria. Participants were screened for study inclusion eligibility and consented online before they completed the survey.

### Data Collection

The online survey was self-administered and included patients’ demographics, clinical characteristics, and eHLQ. The survey was self-administered and took about 15 minutes to complete. The survey was in the field from November to December 2021. More information on the survey using the CHERRIES (The Checklist for Reporting Results of Internet E-Surveys) checklist [33] is presented in Checklist 1.

#### Demographics and Clinical Characteristics

These included patients’ age, sex, ethnicity, race, educational level, marital status, income, clinic distance from residence, residential area, general health status, comorbidities, length of time since being diagnosed with hypertension, number of hypertension medications, number of other medications different from the hypertension medications, BP control status, and the last measured systolic and diastolic BP values.

#### eHealth Literacy

The validated eHLQ [15], a 35-item questionnaire with 7 domains developed from the e-HLF was used to assess e-HL. The first 2 domains provide information on the patient’s capability, the next 3 domains show interaction between the patient and digital services, and the last 2 are about the patient’s experiences with digital services. Each item is rated on a 4-point Likert scale (strongly disagree, disagree, agree, and strongly agree) with the lowest score of 1 and the highest score of 4. The eHLQ was validated using both classical test theory and item response theory psychometrics, and the domain items were found to have strong composite scale reliability [15]. The e-HLQ domain variables include (1) using technology to process health information (5 items, eg, I use technology to find information about health), (2) understanding health concepts and language (5 items, eg, I understand medical results about me), (3) ability to actively engage with digital services (5 items, eg, I can enter data into health technology systems), (4) feel safe and in control (5 items, eg, my electronic health care data are being stored safely), (5) motivated to engage with digital services (5 items, eg, technology helps me take care of my health), (6) access to digital services that work (6 items, eg, I have access to health technology that works), and (7) digital services that suit individual needs (4 items, eg, eHealth systems are provided to me in a way that suits me). Each domain score is the average of the individual item scores. The highest and lowest mean scores possible were 4 and 1, respectively. The higher the score, the better the e-HL status. The eHLQ license was obtained from Swinburne University of Technology.

#### Pilot Testing

The survey was piloted among 12 staff members and graduate student volunteers and then revised for clarity. Another pilot was done through the Qualtrics panel to confirm the content validity and reliability before the final launch.

### Statistical Analysis

Descriptive statistics were used to describe the patient’s demographics and e-HL status. Categorical variables were reported as frequencies (%), while continuous variables were reported as means and SDs. Bivariate analysis using chi-square tests compared patients’ characteristics between RBPM and nonRBPM groups. The reliability and internal consistency of the e-HL domain items were assessed by calculating the Cronbach α. The Mann-Whitney *U* test was used to compare the e-HL domain scores between RBPM and nonRBPM groups. The Mann-Whitney *U* test was used for domain scores comparison because the Shapiro test for normality showed that the data were not normally distributed.

Firth [34,35] logistic regression was used to assess the 7 e-HL domains as the predictors of participation in RBPM. Firth logistic regression uses a penalized likelihood approach to account for any separation in the categorical variables due to the small sample size and reduces bias in the parameter estimates. The outcome variable was participation in RBPM. Independent variables included demographics (age, race, sex, education, marital status, income, clinic distance, residential area, and years of hypertension diagnosis) and the e-HL domains. The independent variables were chosen based on factors already in literature [2,3,36] that could influence people’s use of health services like RBPM. Various regression models were fitted using stepwise forward design and the model with the lowest Akaike information criterion [37] was chosen for the prediction. The final model with the e-HL domains, age, sex, and race was chosen for the prediction because it had the lowest Akaike information criterion value. All analyses were performed using the JJ Allaire R Studio software (version 4.2.1, 2022; Posit, PBC).

### Ethical Considerations

The study was approved by the University of Michigan Institutional Review Board with the approval number HUM00205760. Informed consent and the ability to opt out of the study were provided to the participants, and they consented and signed before they could participate in the study. Anonymized data containing no identifiable personal information were collected from the participants. The participants were compensated with the amount they agreed on with Qualtrics before entering the study. The authors were not privy to the compensation amount.

## Results

### Description of Participants’ Demographics and Clinical Characteristics

A total of 507 people with hypertension meeting the study criteria consented to the study and were surveyed. The mean age for all participants was 60 (SD 14.7) years (Table 1). The respondents were mostly female (306/507, 60.4%), non-Hispanic (483/507, 95.3%), and White (429/507, 84.6%). About two-thirds have some college education or more (339/507, 66.9%), while almost half were married or living as married (243/507, 47.9%). More than half reported having had hypertension for 5 years or more (287/507, 56.6%). Depression or anxiety was the most commonly reported comorbidity (203/507, 40%).

**Table 1.** Participants’ demographics and clinical characteristics.

Variables and categories		All participants (N=507)	RBPM^a^ participation (n=60, 11.8%)	No RBPM participation (n=447, 88.2%)	*P* value
Age (years), mean (SD)		60.09 (14.7)	46.17 (14.71)	61.96 (13.67)	<.001
Age groups (years), n (%)					<.001
Less than 50		83 (16.4)	32 (53.3)	51 (11.4)	
50‐74		318 (62.7)	25 (41.7)	293 (65.5)	
75 and above		106 (20.9)	3 (5)	103 (23)	
Sex, n (%)					.30
Male		201 (39.6)	28 (46.7)	173 (38.7)	
Female		306 (60.4)	32 (53.3)	274 (61.3)	
Ethnicity, n (%)					.09
Hispanic		24 (4.7)	6 (10)	18 (4)	
Non-Hispanic		483 (95.3)	54 (90)	429 (96)	
Race, n (%)					.03
American Indian or Alaska Native		4 (0.8)	2 (3.3)	2 (0.4)	
Asian		7 (1.4)	1 (1.7)	6 (1.3)	
Black or African American		61 (12)	12 (20)	49 (11.0)	
White		429 (84.6)	45 (75)	384 (85.9)	
Other		6 (1.2)	0 (0)	6 (1.3)	
Education level, n (%)					.05
Less than high school		15 (3)	2 (3.3)	13 (2.9)	
High school graduate		153 (30.2)	17 (28.3)	136 (30.4)	
Some college		176 (34.7)	13 (21.7)	163 (36.5)	
Bachelor’s		148 (29.2)	24 (40)	124 (27.7)	
Graduate or Prof degree		15 (3)	4 (6.7)	11 (2.5)	
Marital status, n (%)					.003
Single		86 (17)	14 (23.3)	72 (16.1)	
Married		207 (40.8)	32 (53.3)	175 (39.1)	
Living as married		36 (7.1)	7 (11.7)	29 (6.5)	
Separated		18 (3.6)	2 (3.3)	16 (3.6)	
Divorced		94 (18.5)	3 (5)	91 (20.4)	
Widowed		66 (13)	2 (3.3)	64 (14.3)	
Annual household income, n (%)					>.99
Less than US $20,001		77 (15.2)	9 (15)	68 (15.2)	
US $20,001 to US $35,000		120 (23.7)	15 (25)	105 (23.5)	
US $35,001 to US $50,000		94 (18.5)	11 (18.3)	83 (18.6)	
US $50,001 to US $75,000		99 (19.5)	11 (18.3)	88 (19.7)	
US $75,001 or more		106 (20.9)	13 (21.7)	93 (20.8)	
Prefer not to say		11 (2.2)	1 (1.7)	10 (2.2)	
Clinic distance (miles), n (%)					.04
Less than 5		204 (40.2)	19 (31.7)	185 (41.4)	
Between 5 and 10		194 (38.3)	32 (53.3)	162 (36.2)	
More than 10		109 (21.5)	9 (15)	100 (22.4)	
Area, n (%)					.01
Urban		130 (25.6)	24 (40)	106 (23.7)	
Suburban		245 (48.3)	22 (36.7)	223 (49.9)	
Exurban		15 (3)	1 (1.7)	14 (3.1)	
Rural		104 (20.5)	9 (15)	95 (21.3)	
Blank answer		13 (2.6)	4 (6.7)	9 (2)	
General health status, n (%)					.10
Poor		22 (4.3)	1 (1.7)	21 (4.7)	
Fair		120 (23.7)	11 (18.3)	109 (24.4)	
Good		238 (46.9)	26 (43.3)	212 (47.4)	
Very good		113 (22.3)	18 (30)	95 (21.3)	
Excellent		14 (2.8)	4 (6.7)	10 (2.2)	
Comorbidity, n (%)					.01
Heart condition		0 (0)	0 (0)	0 (0)	
Diabetes		128 (25.2)	18 (30)	110 (24.6)	
Depression or anxiety		203 (40)	35 (58.3)	168 (37.6)	
Chronic kidney disease		24 (4.7)	1 (1.7)	23 (5.1)	
Other diseases		99 (19.5)	8 (13.3)	91 (20.4)	
No comorbidity		137 (27)	7 (11.7)	130 (29.1)	
Hypertension history, n (%)					<.001
Less than 1 year		22 (4.3)	0 (0)	22 (4.9)	
1 year to less than 2 years		44 (8.7)	16 (26.7)	28 (6.3)	
2 years to less than 3 years		63 (12.4)	15 (25)	48 (10.7)	
3 years to less than 4 years		47 (9.3)	10 (16.7)	37 (8.3)	
4 years to less than 5 years		44 (8.7)	4 (6.7)	40 (8.9)	
5 years or more		287 (56.6)	15 (25)	272 (60.9)	

a RBPM: remote blood pressure monitoring.

A total of 60 respondents out of 507 (11.8%) reported participation in RBPM (Table 1). The RBPM participation group had a significantly lower age (mean 46.2, SD 14.7 years) than the nonRBPM participation group (mean 62, SD 13.7 years), *P*<.001. The RBPM participation group also had more people in the married category (32/60, 53.3% vs 175/447, 39.1%). The majority (45/60, 75.1%) of those participating in RBPM reported less than 5 years since diagnosis of hypertension compared to 39.1% (175/447) in the nonRBPM group (Table 1).

### eHealth Literacy

The calculated Cronbach α for our sample for the eHLQ domains 1 to 7 ranged from 0.77 to 0.82. The respondents reported e-HL mean scores above 2 (which is half of the highest possible score) in all the 7 domains of the eHLQ (Table 2). However, the RBPM participating group had significantly higher e-HL mean scores than the nonRBPM group in all 7 domains.

**Table 2.** Mean scores for each eHealth literacy domain (comparison was performed using Mann-Whitney *U* test).

e-HL^a^ domains	e-HL domains’ Cronbach α (95% CI)	All participants (N=507) mean (SD)	RBPM^b^ participation (n=60, 11.8%), mean (SD)	No RBPM participation (n=447, 88.2%), mean (SD)	*P* value
Using technology to process health information	0.80 (0.76-0.83)	2.96 (0.56)	3.29 (0.55)	2.92 (0.55)	<.001
Understanding health concepts and language	0.77 (0.73-0.80)	3.11 (0.46)	3.28 (0.51)	3.09 (0.44)	.001
Ability to actively engage with digital services	0.82 (0.79-0.85)	3.00 (0.60)	3.27 (0.56)	2.96 (0.59)	<.001
Feel safe and in control	0.80 (0.77-0.84)	3.09 (0.52)	3.33 (0.52)	3.06 (0.51)	<.001
Motivated to engage with digital services	0.81 (0.77-0.84)	3.01 (0.55)	3.29 (0.51)	2.97 (0.55)	<.001
Access to digital services that work	0.81 (0.77-0.83)	3.08 (0.48)	3.34 (0.47)	3.05 (0.47)	<.001
Digital services that suit individual need	0.80 (0.75-0.83)	2.89 (0.61)	3.30 (0.60)	2.83 (0.59)	<.001

a e-HL: eHealth literacy.

b RBPM: remote blood pressure monitoring.

### e-HL Domain Predictors of RBPM Participation

The univariate regression of each e-HL domain with RBPM participation yielded a significantly positive association with RBPM participation. The unadjusted odds ratios (ORs) include (1) using technology to process health information (OR 3.62, 95% CI 2.15‐6.23; *P*<.001), (2) understanding health concepts and language (OR 2.54, 95% CI 1.41‐4.62; *P*=.001), (3) ability to actively engage with digital services (OR 2.54, 95% CI 1.56‐4.24; *P*<.001), (4) feel safe and in control (OR 2.81, 95% CI 1.64‐4.91; *P*<.001), (5) motivated to engage with digital services (OR 3.09, 95% CI 1.82‐5.37; *P*<.001), (6) access to digital services that work (OR 3.69, 95% CI 2.07‐6.71; *P*<.001), and (7) digital services that suit individual needs (OR 4.18, 95% CI 2.52‐7.15; *P*<.001).

When participation in RBPM was regressed with all the 7 domains of e-HLQ together as variables, higher scores on having digital services that suit individual needs (domain 7) were associated with higher odds of RBPM participation (adjusted odds ratio [aOR] 4.49, 95% CI 1.65‐13.28; *P*=.003) (Multimedia Appendix 1). The other 6 domains were not statistically significant predictors. Adjusting the model with a single demographic variable at a time resulted in domain 7 as the only significant predictor of RBPM participation (Multimedia Appendix 2). Domain 7 remained the only significant e-HL domain with the model adjustment with age, sex, and race simultaneously (Table 3). Holding age, sex, and race constant, every unit increase in domain 7 mean scores resulted in 184% higher odds of participation in RBPM. Further adjustment of the model with additional demographics (education, marital status, income, clinic distance, residential area, and years of hypertension diagnosis) resulted in the loss of any statistically significant association between the e-HL domains and participation in RBPM (Multimedia Appendix 3).

**Table 3.** Firth logistic regression of remote blood pressure monitoring participation with eHealth literacy domains adjusted for age, sex, and race.

Predictor variables	Adjusted odds ratio (95% CI)
Using technology to process health information	1.121 (0.372-4.805)
Understanding health concepts and language	0.692 (0.242-2.004)
Ability to actively engage with digital services	0.615 (0.219-1.688)
Feel safe and in control	1.167 (0.483-2.931)
Motivated to engage with digital services	0.627 (0.184-2.110)
Access to digital services that work	1.780 (0.521-6.151)
Digital services that suit individual needs	2.836 (1.002-8.837)

## Discussion

### Principal Findings

e-HL is a key resource for engagement in any digital health services. However, e-HL is not often assessed in telemedicine studies [38]. This study is the first to our knowledge to use the eHLQ to assess participation in RBPM. Participants in the RBPM group had higher e-HL mean scores in all 7 domains than those in the nonRBPM group. Thus, underscoring that digital skills and knowledge may enhance digital services use.

However, having digital services that suit individual needs (domain 7) was the only significant predictor of RBPM participation when considering all 7 domains of the e-HL framework together. Interestingly, domain 7 remained a significant predictor of RBPM participation after accounting for age, sex, and race simultaneously. These results suggest that although it is imperative for a patient to know about one’s health, be motivated and actively engaged in digital services, and have a sense of safety and access to digital services that work, if an RBPM digital service does not fit the individual needs of the patient, the likelihood of taking part in RBPM is greatly reduced. And having suitable RBPM services is important regardless of the patient’s age, sex, or race.

Personalization of technology interventions has been highlighted as a crucial strategy to improve patients’ engagement and adherence to digital health services [36,39-42]. It is, therefore, necessary to tailor RBPM services to individual patients by considering, for example, what frequency and time of self-BP measurement is suitable for the patient, what mode of electronic transmission of self-measured BP works best for the patient, and what degree of feedback is needed by the patient and the communication channel preferred by the patient to get the feedback among other things [39,42]. Personalization of RBPM services would also help in ensuring that only required data are obtained from the patient, reducing effort and time spent in reviewing unnecessary data by the health care providers.

Our study is uniquely focused on current BP telemonitoring participation, making it difficult to compare our findings to previous studies that reported willingness or readiness to participate in BP telemonitoring [23,43]. Moreover, it is the first quantitative study on RBPM using the robustness of eHLQ based on the e-HL framework to assess RBPM participation among adults with HTN.

The RBPM participants in our study were younger, more likely to be married, and have less than 5 years since hypertension diagnosis. This could be a result of health providers’ bias in RBPM participation enrollment or some other reasons. Further studies involving patients with hypertension, health care providers, and clinic administrations are needed to understand why RBPM participation population were not older, single, and with longer years of hypertension diagnosis.

### Study Limitations

This study is limited by its cross-sectional design, use of a self-administered electronic survey, fluency in English criterion, having mostly non-Hispanic White participants with hypertension, and small number of RBPM participants. We recruited from Qualtrics panel members who share some demographic differences (eg, sex, ethnicity, and percentage with BP control) and similarities (income and education) with the random sample of US adults with hypertension reported in the 2017‐2018 National Health and Nutrition Examination Survey (NHANES) [44]. However, we believe that the large sample and robustness of our analytical approach provide a reasonable estimate of the relationship between the factors identified. Recruiting participant representatives from all age brackets and educational levels helped reduce age and education biases.

### Conclusions

Higher e-HL status among patients with hypertension would increase RBPM participation. RBPM services should be personalized by providing patients with suitable telemonitoring infrastructures and technical support to maximize individual benefits.

## Supplementary material

10.2196/71926 Multimedia Appendix 1 Firth logistic regression of remote blood pressure monitoring participation with eHealth literacy only.

10.2196/71926 Multimedia Appendix 2 Firth logistic regression of remote blood pressure monitoring participation with eHealth literacy domains and single demographic adjustment at a time.

10.2196/71926 Multimedia Appendix 3 Firth logistic regression of remote blood pressure monitoring with electronic health literacy domains adjusted for age, sex, race, income, education, marital status, clinic distance, residential area, and years since hypertension diagnosis.

10.2196/71926 Checklist 1 CHERRIES checklist.

## Abbreviations

BP: blood pressure
CHERRIES: Checklist for Reporting Results of Internet E-Surveys
e-HL: eHealth literacy
e-HLF: eHealth literacy framework
eHEALS: eHealth Literacy Scale
eHLQ: eHealth literacy questionnaire
HINTS: Health Information National Trends Survey
NHANES: National Health and Nutrition Examination Survey
OR: odds ratio
RBPM: remote blood pressure monitoring
