# Supplementary material for: eHealth Literacy and Participation in Remote Blood Pressure Monitoring Among Patients With Hypertension: Cross-Sectional Study
Source: J Med Internet Res. 2025 Jul 31;27:e71926. doi: 10.2196/71926 (PMC12314467; doi:10.2196/71926)
Supplement: Multimedia Appendix 1 [file jmir-v27-e71926-s001.doc]

**Table S1.**

| Predictor variables | Odds ratio (95% CI) |  |
| --- | --- | --- |
| 1. Using technology to process health information | 2.45 (0.71-8.78) |  |
| 2. Understanding health concepts and language | 0.58 (0.22-1.60) |  |
| 3. Ability to actively engage with digital services | 0.84 (0.33-2.15) |  |
| 4. Feel safe and in control | 1.13 (0.49-2.73) |  |
| 5. Motivated to engage with digital services | 0.50 (0.15-1.73) |  |
| 6. Access to digital services that work | 1.14 (0.35-3.78) |  |
| 7. Digital services that suit individual needs | 4.49 (1.65-13.28) |  |
