# Supplementary material for: eHealth Literacy and Participation in Remote Blood Pressure Monitoring Among Patients With Hypertension: Cross-Sectional Study
Source: J Med Internet Res. 2025 Jul 31;27:e71926. doi: 10.2196/71926 (PMC12314467; doi:10.2196/71926)
Supplement: Multimedia Appendix 2 [file jmir-v27-e71926-s002.doc]

**Table S1.**

| Predictor variables | Adjusted odds ratio (95% CI) |
| --- | --- |
| 1.Using technology to process health information | 1.222 (0.323-4.730) |
| 2. Understanding health concepts and language | 0.626 (0.205-1.890) |
| 3. Ability to actively engage with digital services | 0.598 (0.203-1.704) |
| 4. Feel safe and in control | 1.188 (0.462-3.292) |
| 5. Motivated to engage with digital services | 0.792 (0.183-3.406) |
| 6. Access to digital services that work | 2.234 (0.605-8.164) |
| 7. Digital services that suit individual needs | 1.975 (0.653-6.619) |
