# Supplementary material for: eHealth Literacy and Participation in Remote Blood Pressure Monitoring Among Patients With Hypertension: Cross-Sectional Study
Source: J Med Internet Res. 2025 Jul 31;27:e71926. doi: 10.2196/71926 (PMC12314467; doi:10.2196/71926)
Supplement: Multimedia Appendix 3 [file jmir-v27-e71926-s003.doc]

**Table S1.**

| e-HL^a^ domain predictors | Age,  OR^b^ (95% CI) | Sex, OR (95% CI) | Race, OR (95% CI) | Education OR (95% C1) | Marital status, OR (95% CI) | Income, OR (95% CI) | Area of residence, OR (95% CI) | Clinic distance, OR (95% CI) | Years of HTN^c^, OR (95% CI) |
| --- | --- | --- | --- | --- | --- | --- | --- | --- | --- |
| 1. Using technology to process health information | 1.261 (0.362-4.553) | 2.528 (0.724-9.127) | 2.397 (0.691-8.617) | 2.596 (0.735-9.510) | 2.573 (0.736-9.211) | 2.453 (0.707-8.806) | 1.636 (0.457-6.155) | 2.067 (0.595-7.435) | 1.942 (0.549-7.094) |
| 2. Understanding health concepts and language | 0.689 (0.243-1.979) | 0.572 (0.210-1.570) | 0.601 (0.221-1.646) | 0.537 (0.194-1.495) | 0.562 (0.204-1.560) | 0.584 (0.214-1.606) | 0.509 (0.182-1.438) | 0.619 (0.226-1.702) | 0.628 (0.227-1.740) |
| 3. Ability to actively engage with digital services | 0.647 (0.230-1.777) | 0.800 (0.309-2.038) | 0.842 (0.323-2.157) | 0.789 (0.302-2.032) | 0.823 (0.320-2.074) | 0.827 (0.315-2.138) | 0.824 (0.312-2.147) | 0.795 (0.307-2.030) | 0.748 (0.277-1.968) |
| 4. Feel safe and in control | 1.178 (0.489-2.956) | 1.115 (0.480-2.694) | 1.076 (0.463-2.610) | 1.206 (0.519-2.934) | 1.143 (0.488-2.797) | 1.171 (0.500-2.871) | 1.075 (0.452-2.698) | 1.066 (0.457-2.598) | 1.305 (0.553-3.237) |
| 5. Motivated to engage with digital services | 0.618 (0.180-2.099) | 0.495 (0.148-1.699) | 0.502 (0.148-1.738) | 0.472 (0.139-1.659) | 0.421 (0.123-1.459) | 0.486 (0.143-1.698) | 0.900 (0.233-3.519) | 0.469 (0.136-1.647) | 0.553 (0.162-1.927) |
| 6. Access to digital services that work | 1.752 (0.505-6.100) | 1.164 (0.365-3.805) | 1.209 (0.368-4.056) | 1.296 (0.389-4.373) | 1.217 (0.381-3.958) | 1.156 (0.347-3.916) | 1.327 (0.381-4.675) | 1.348 (0.407-4.508) | 1.373 (0.403-4.742) |
| 7. Digital services that suit individual needs | 2.847 (1.003-8.888) | 4.570 (1.680-13.539) | 4.263 (1.563. 12.650) | 4.200 (1.536-12.414) | 4.325 (1.606-12.715) | 4.485 (1.638-13.388) | 3.734 (1.349-11.317) | 4.852 (1.736-14.751) | 3.328 (1.163-10.279) |

^a^e-HL: eHealth literacy.

^b^OR: odds ratio.

^c^HTN: hypertension.
